# Mounting Challenges: A Tragic Case of Rheumatoid Arthritis-Associated Interstitial Lung Disease in an Incarcerated Patient, Highlighting Healthcare Access Barriers in Correctional Settings

**DOI:** 10.7759/cureus.102545

**Published:** 2026-01-29

**Authors:** Shaivya Pathak

**Affiliations:** 1 Internal Medicine, East Carolina University, Greenville, USA

**Keywords:** biologic therapy, correctional healthcare, healthcare disparities, health policy, incarceration, interstitial lung disease, rheumatoid arthritis, tofacitinib

## Abstract

Rheumatoid Arthritis-associated Interstitial Lung Disease (RA-ILD) is a serious extra-articular manifestation associated with significant morbidity and mortality. We present the case of a 48-year-old incarcerated male with seropositive rheumatoid arthritis who was clinically stable on tofacitinib and methotrexate for over one year. Following the discontinuation of tofacitinib, which the patient attributed to correctional facility formulary changes and restrictions, he developed progressive respiratory symptoms. He presented to the hospital two months later with acute hypoxic respiratory failure necessitating intubation. Computed tomography of his lungs revealed extensive ground-glass opacities with honeycombing consistent with usual interstitial pneumonia pattern ILD, a condition previously undiagnosed in this patient. Despite aggressive management including pulse-dose corticosteroids, bronchoscopy, empiric antimicrobial treatment and comprehensive infectious workup, he continued to deteriorate over an eight-day intensive care unit course. He was deemed not a candidate for extracorporeal membrane oxygenation or lung transplantation due to acute illness severity, and the family eventually elected to transition to comfort care. This case highlights the profound challenges of managing rheumatoid arthritis in incarcerated populations, including barriers to accessing expensive targeted therapies, limited specialist availability, and the importance of treatment continuity for patients with complex autoimmune diseases. We discuss the practical implications of these healthcare disparities and advocate for policy reforms to ensure adequate rheumatologic care in correctional settings.

## Introduction

Rheumatoid arthritis (RA) is a chronic, progressive autoimmune disease affecting an estimated 1.36 million adults in the United States [1]. Beyond joint manifestations, RA is associated with significant extra-articular complications, with interstitial lung disease (ILD) representing one of the most serious ones. RA-ILD affects approximately 10-30% of patients with RA and is associated with a median survival of only 2.6 years following diagnosis [2]. The usual interstitial pneumonia (UIP) pattern, characterized by honeycombing and traction bronchiectasis, carries a particularly poor prognosis.

The management of RA has been revolutionized by the introduction of biologic and targeted synthetic disease-modifying antirheumatic drugs (b/tsDMARDs), which have demonstrated efficacy in controlling disease activity and potentially stabilizing or improving pulmonary manifestations [3]. However, these advanced therapies come with substantial costs, with annual expenditures ranging from $43,935 to $101,402 for commercially insured patients [4]. This financial burden creates unique challenges in correctional healthcare settings, where approximately 15% of inmates have arthritis or other rheumatic diseases [5].

The United States incarcerates approximately 1.9 million individuals, with correctional facilities constitutionally obligated under the Eighth Amendment to provide adequate medical care [6]. However, the definition of adequate remains contested, particularly regarding expensive speciality medications. Many correctional facilities maintain strict formularies that restrict access to costly treatments, often requiring patients to fail multiple less expensive options before qualifying for advanced therapies [5]. This case report describes a patient with RA who experienced a fatal ILD exacerbation following the discontinuation of his targeted therapy, and examines the broader implications of healthcare access barriers in correctional settings.

## Case presentation

A 48-year-old incarcerated male with a history of seropositive rheumatoid arthritis diagnosed five years prior presented to the hospital with acute hypoxic respiratory failure. His RA was characterized by high-titer rheumatoid factor and anti-cyclic citrullinated peptide (anti-CCP) antibodies greater than 250 U/mL, with a strong family history including his mother, maternal grandmother, maternal uncle, and brother. He had a 30-pack-year smoking history but had quit before his RA diagnosis.

His treatment history included meloxicam (ineffective), adalimumab (ineffective), and methylprednisolone (associated with weight gain). He was subsequently started on tofacitinib 5 mg twice daily by his community rheumatologist, with significant improvement. At his most recent rheumatology evaluation, seven months before hospital presentation, performed via telehealth through the correctional system, he had been on tofacitinib for approximately 15 months and reported minimal symptoms. Physical examination revealed ulnar deviation of the fingers (left greater than right) and swan neck deformities. His Clinical Disease Activity Index (CDAI) was 11, indicating moderate disease activity at the treatment goal [7]. At that visit, methotrexate 15 mg weekly was added, given residual morning stiffness and hand tenderness, with plans for further joint imaging and routine follow-up. Approximately two months before hospitalization, tofacitinib was discontinued. The patient attributed this to formulary changes within the correctional facility, though this could not be independently verified. The patient subsequently developed progressive shortness of breath and a non-productive cough over three weeks. He was initially treated at the correctional facility with a five-day course of prednisone and guaifenesin without improvement. He was then transferred to a hospital for worsening shortness of breath associated with hypoxia.

**Figure 1:**
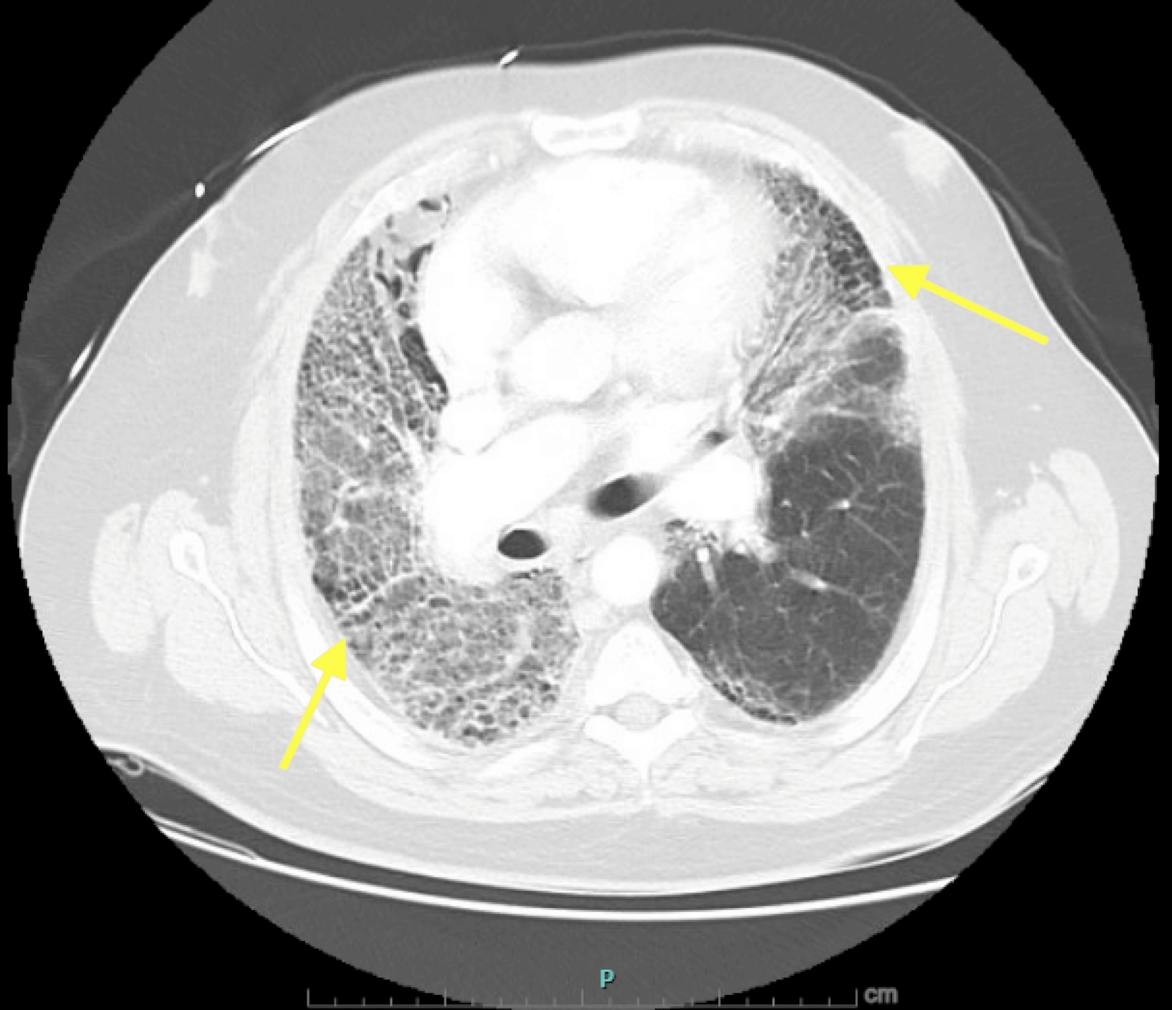
CTA of the chest demonstrating extensive bilateral ground-glass opacities. The image demonstrates extensive bilateral ground-glass opacities (yellow arrows), right greater than left, with scattered areas of honeycombing. CTA: computed tomography angiography.

Computed tomography angiography (CTA) of the chest revealed extensive ground-glass interstitial opacities in both lungs (right greater than left) with scattered areas of honeycombing, compatible with chronic interstitial lung disease in a usual interstitial pneumonia (UIP) pattern (Figure 1).

There was no evidence of pulmonary embolism. Laboratory studies demonstrated leukocytosis (17,890/µL) with marked peripheral eosinophilia (11%, absolute count 1,270/µL), elevated inflammatory markers (ESR and CRP), and a negative respiratory viral panel including COVID-19 and influenza (Table 1).

**Table 1:** Pertinent Laboratory Values. H: high; L: low; BAL: bronchoalveolar lavage; PJP: *pneumocystis jirovecii pneumonia*; GFR: glomerular filtration rate; ESR: erythrocyte sedimentation rate; LDH: lactate dehydrogenase. *Indeterminate due to absent mitogen response (Mitogen-Nil 0.00).

Test	Reference Range	Result (Peak/Nadir)
Hematology		
White Blood Cell Count	4.5-11.0 x10³/µL	14.06 x10³/µL (H)
Hemoglobin	13.5-17.5 g/dL	8.3 g/dL (L)
Platelet Count	150-450 x10³/µL	94 x10³/µL (L)
Absolute Eosinophils (Admission)	0-0.5 x10³/µL	1.27 x10³/µL (H)
Absolute Lymphocytes	1.0-4.8 x10³/µL	0.25 x10³/µL (L)
Chemistry		
Sodium	136-145 mmol/L	153 mmol/L (H)
Blood Urea Nitrogen	7-20 mg/dL	95 mg/dL (H)
Creatinine (Baseline → Peak)	0.7-1.3 mg/dL	0.96 → 2.27 mg/dL (H)
GFR (Baseline → Nadir)	> 60 mL/min/1.73m²	98 → 35 mL/min/1.73m² (L)
Albumin	3.5-5.0 g/dL	2.6 g/dL (L)
Globulin	2.0-3.5 g/dL	5.7 g/dL (H)
Inflammatory Markers		
C-Reactive Protein	< 0.5 mg/dL	253.7 mg/dL (H)
ESR	0-20 mm/hr	124 mm/hr (H)
LDH	140-280 U/L	474 U/L (H)
Arterial Blood Gas (on 100% FiO2)		
pH	7.35-7.45	7.26 (L)
pCO_2_	35-45 mmHg	65 mmHg (H)
pO_2_	80-100 mmHg	77-164 mmHg
Autoimmune Studies		
Anti-CCP Antibody	< 20 U/mL	> 250 U/mL (H)
Rheumatoid Factor (IgG)	< 20 IU/mL	> 100 IU/mL (H)
Rheumatoid Factor (IgA)	< 20 IU/mL	> 100 IU/mL (H)
Rheumatoid Factor (IgM)	< 20 IU/mL	> 100 IU/mL (H)
ANA (HEp-2 Substrate)	< 1:80	< 1:80 (Negative)
IgE	< 100 IU/mL	453 IU/mL (H)
IgM	40-230 mg/dL	279 mg/dL (H)
IgG	700-1600 mg/dL	1,301 mg/dL
IgA	70-400 mg/dL	225 mg/dL
Complement Studies		
CH50 (Total Complement)	30-75 U/mL	25 U/mL (L)
C3 Complement	90-180 mg/dL	117 mg/dL
C4 Complement	10-40 mg/dL	22 mg/dL
Infectious Workup - Respiratory		
SARS-CoV-2 PCR	Not Detected	Not Detected
Influenza A PCR	Not Detected	Not Detected
Influenza B PCR	Not Detected	Not Detected
Respiratory Syncytial Virus	Not Detected	Not Detected
*Streptococcus pneumoniae A*ntigen	Negative	Negative
Legionella Urinary Antigen	Negative	Negative
Infectious Workup-Fungal/Mycobacterial		
Beta-D-Glucan (Fungitell)	< 60 pg/mL	< 31 pg/mL
Aspergillus Galactomannan Antigen	< 0.5 Index	< 0.500 Index
Aspergillus fumigatus IgG Antibody	≤ 102 mg/L	12.9 mg/L
Histoplasma/Blastomyces Antigen	Not Detected	Not Detected
Coccidioides Antibody Screen	Negative	Negative
QuantiFERON-TB Gold Plus	Negative	Indeterminate*
BAL AFB Smear	No Organisms Seen	Negative
BAL AFB Culture	No Growth	No Growth
BAL Fungal Culture	No Growth	No Growth
Infectious Workup - Viral/Other		
HIV 1/2 Antibody	Negative	Negative
HIV P24 Antigen	Negative	Negative
Hepatitis B Surface Antigen	Non-Reactive	Non-Reactive
Hepatitis B Core Antibody	Non-Reactive	Non-Reactive
Hepatitis C Antibody	Non-Reactive	Non-Reactive
CMV DNA Quantitative	Not Detected	0.0 (Not Detected)
EBV DNA Quantitative	Not Detected	0.0 (Not Detected)
Cultures		
Blood Culture (Aerobic/Anaerobic)	No Growth	No Growth
Urine Culture	No Growth	No Growth
Respiratory Culture	No Growth	No Growth
Quantitative BAL Culture	No Growth	No Growth
MRSA Nasal Screen	Not Detected	Not Detected
Bronchoalveolar Lavage Studies		
BAL Gram Stain	No Organisms	No Organisms
PJP PCR (BAL from LLL)	Not Detected	Negative
PJP Cytopathology	Negative	Negative
GMS Stain for Pneumocystis	Negative	No Pneumocystis Identified
BAL Cytology	Negative	Negative for Malignancy

The patient was placed on bilevel positive airway pressure (BiPAP) but failed non-invasive ventilation after 48 hours, requiring endotracheal intubation and mechanical ventilation. He received one dose of methylprednisolone 125 mg along with empiric intravenous antibiotics, which included ceftriaxone, azithromycin, piperacillin-tazobactam, and vancomycin. An extensive infectious workup was pursued. Bronchoscopy with bronchoalveolar lavage (BAL) on hospital day four revealed minimal clear mucoid secretions with mild airway hyperemia but no significant findings. BAL studies, including bacterial, fungal, and mycobacterial cultures, were negative. *Pneumocystis jirovecii pneumonia* (PJP) cytopathology and PCR were negative. Beta-D-glucan (Fungitell), *aspergillus galactomannan*, *coccidioides* serology, HIV antibody, hepatitis panel, Epstein-Barr virus, and cytomegalovirus studies were all negative. QuantiFERON-TB Gold testing was indeterminate. Repeat connective tissue disease workup confirmed seropositive RA with anti-CCP greater than 250 U/mL and positive rheumatoid factor (Table 1).

**Figure 2:**
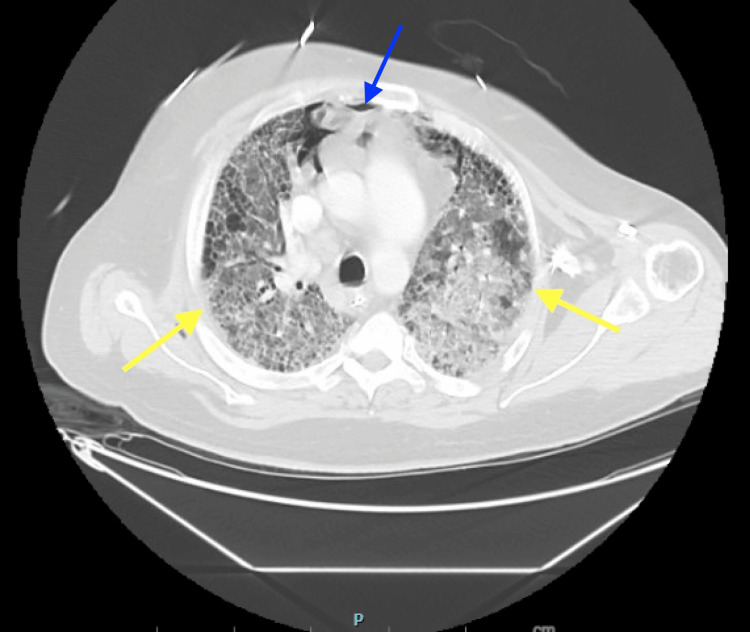
Interval CT of the chest. The image shows significant progression of interstitial lung disease with peripheral honeycombing, paraseptal emphysema, worsening bilateral ground-glass opacities (yellow arrows), and pneumomediastinum (blue arrow). CT: computed tomography.

**Figure 3:**
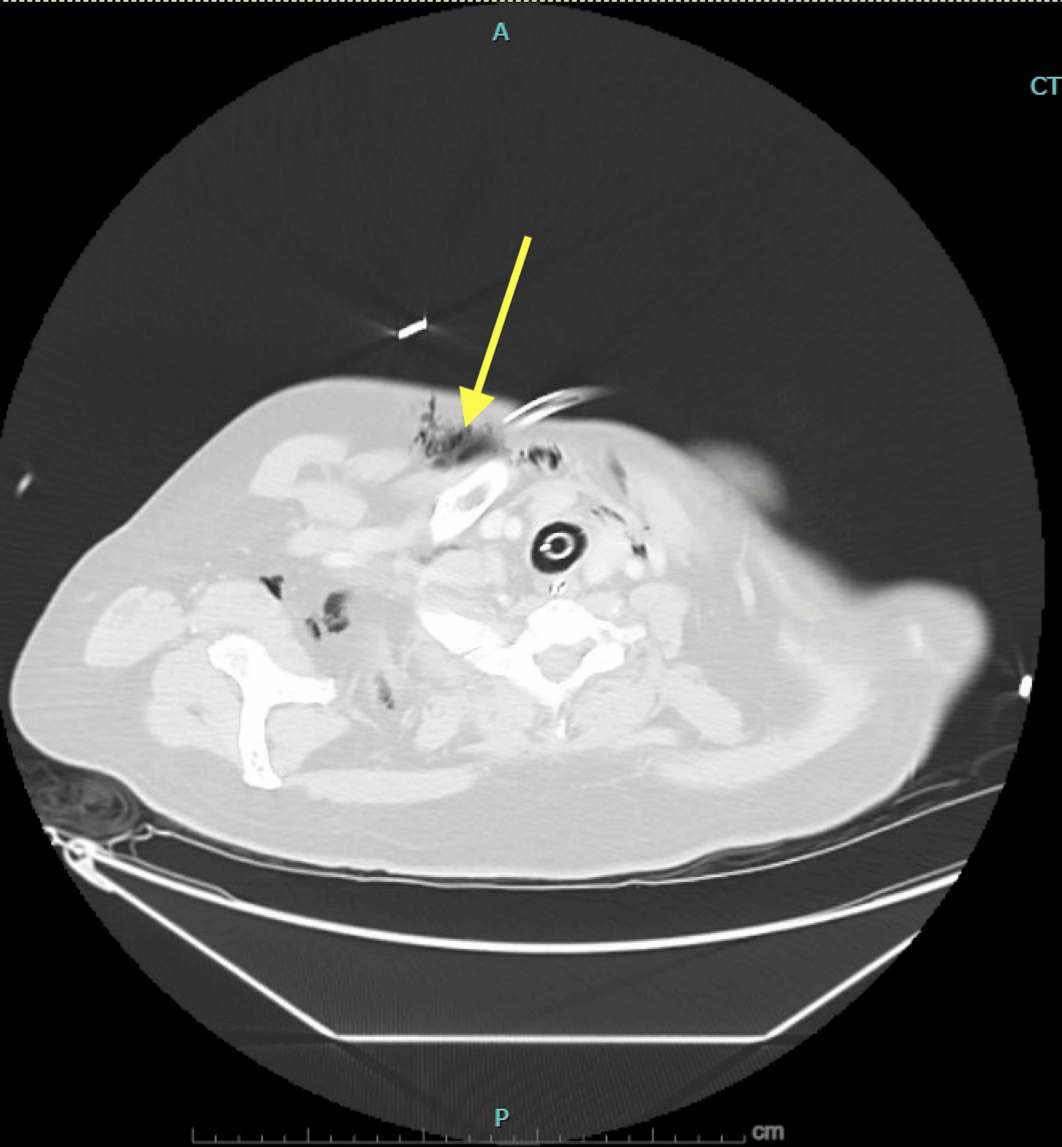
CT of the chest demonstrating subcutaneous emphysema. The image demonstrates subcutaneous emphysema in the anterior chest wall (yellow arrow). CT: computed tomography.

The patient was treated with pulse-dose methylprednisolone (500 mg daily for three days) followed by maintenance methylprednisolone 80 mg daily for suspected RA-ILD exacerbation. Atovaquone was initiated for PJP prophylaxis, given high-dose corticosteroid therapy. Initial ventilator management required airway pressure release ventilation (APRV) with high FiO_2_ requirements (90-100%). By hospital day six, FiO_2_ requirements improved to 50%. However, the patient developed acute kidney injury (creatinine rising from 0.84 to 2.93 mg/dL), attributed to a combination of hypotension and high mean airway pressures. On hospital day seven, he developed fever, worsening hypoxia, and new pneumomediastinum. Repeat CT chest demonstrated significantly worsening ground-glass opacities bilaterally with moderate pneumomediastinum and subcutaneous emphysema (Figures 2,3). 

**Figure 4:**
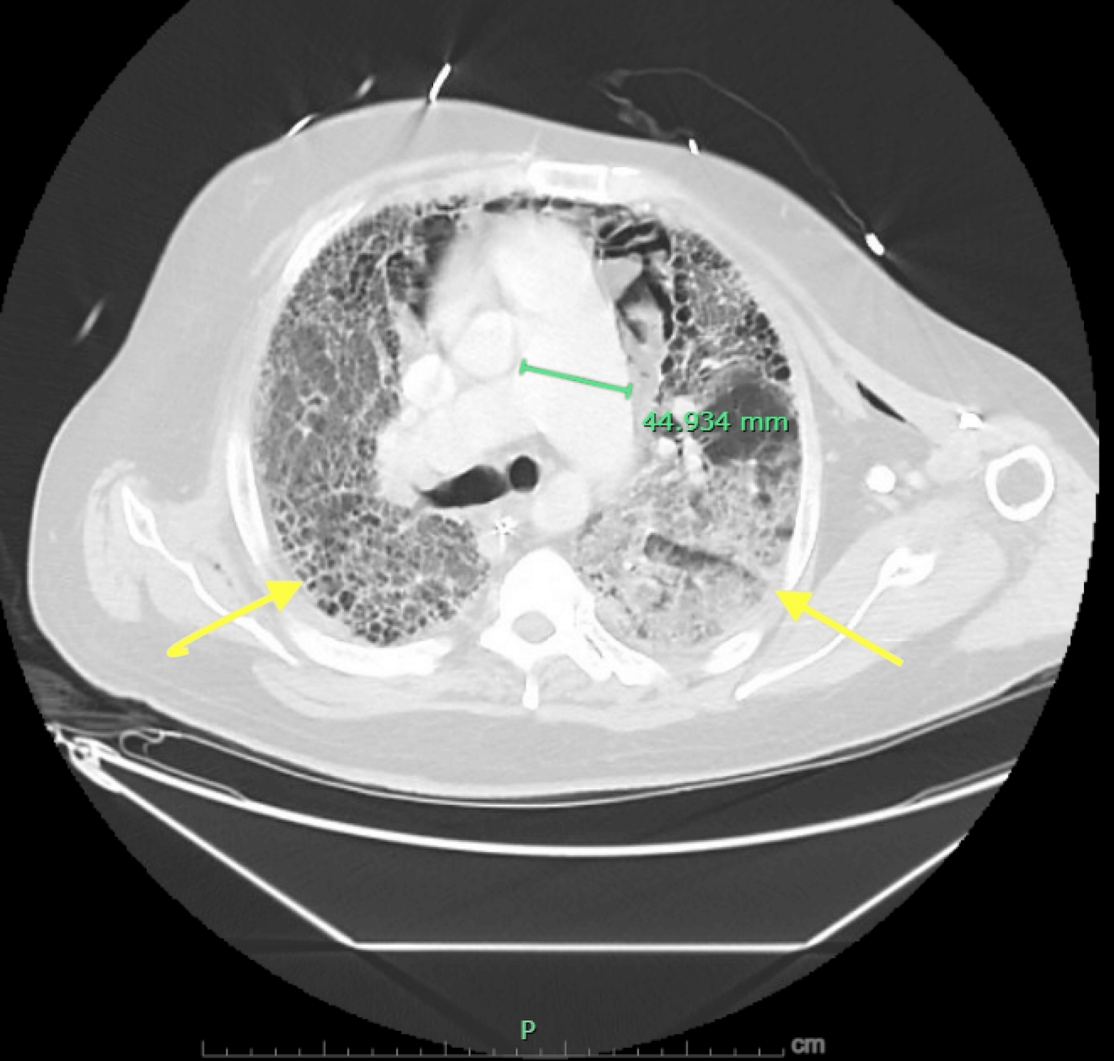
CT of the chest demonstrating extensive bilateral ground-glass opacities. The image demonstrates extensive bilateral ground-glass opacities (yellow arrows) with honeycombing. Note dilated main pulmonary artery measuring 44.9 mm, suggestive of pulmonary hypertension. CT: computed tomography.

The pulmonary artery was dilated, suggesting pulmonary hypertension (Figure 4). Broad-spectrum antibiotics were reinitiated empirically, though repeat BAL on hospital day eight again demonstrated no infectious aetiology. The patient required neuromuscular blockade and prone positioning to optimize ventilation. Inhaled epoprostenol was initiated for pulmonary hypertension. Despite these measures, he remained on 100% FiO_2_ with marginal oxygen saturations.

Given the patient's refractory hypoxemic respiratory failure, extracorporeal membrane oxygenation (ECMO) and lung transplantation were considered. The cardiac intensive care unit was consulted for ECMO evaluation. Multiple extensive conference calls were held with transplant teams at two quaternary academic medical centres. Ultimately, the patient was not deemed a candidate for either intervention due to the severity of his acute illness, including multi-organ dysfunction, the irreversibility of his underlying ILD, and the lack of prior pre-transplant evaluation. While his incarcerated status and the subsequent challenges of ensuring necessary post-transplant follow-up were noted in discussions, the primary barriers were clinical rather than social. After careful deliberation, the family elected to transition this patient to comfort-focused care. The patient was made do-not-resuscitate, and plans were made for compassionate extubation. He died shortly thereafter with family present.

## Discussion

This case illustrates the convergence of a serious rheumatologic complication and the systemic challenges of providing adequate care to incarcerated individuals with chronic autoimmune diseases. While we cannot establish with certainty that continued tofacitinib therapy would have prevented this outcome, the temporal relationship between drug discontinuation and clinical deterioration is notable, and emerging evidence suggests that JAK inhibitors may have a role in stabilizing or improving RA-ILD [8].

The burden of RA in incarcerated populations

The United States incarcerates more people per capita than any other nation, with approximately 1.9 million individuals currently in prisons and jails [9]. Studies indicate that 15% of inmates in state and federal prisons have arthritis or other rheumatic diseases, and this proportion is expected to increase as the incarcerated population ages [5]. Current estimates indicate that 12% of incarcerated individuals are older than 55 years, representing a 300% increase over the past two decades [5].

Rheumatologic care presents unique challenges in correctional settings. Access to rheumatologists is limited by a nationwide shortage of specialists, particularly in rural areas where many prisons are located [10]. A 2015 study found that only 3.9% of adult rheumatologists practised in the Southwest, while 21% were concentrated in the Northeast [11]. This geographic maldistribution means that incarcerated individuals requiring rheumatologic care face prolonged wait times or may have no access until transferred to facilities with better healthcare infrastructure. While telemedicine has emerged as a valuable tool for delivering healthcare in corrections, its utility in rheumatology is limited by the need for physical examination to accurately assess joint and pleural inflammation [5].

The cost barrier

Perhaps the most significant barrier to adequate RA care in correctional settings is medication cost. While conventional synthetic DMARDs such as methotrexate can cost as little as $16 per month, biologic and targeted synthetic DMARDs range from $43,935 to $101,402 annually [4,12]. National survey data from 2018-2020 demonstrated that prescription medications account for 41% of total healthcare expenditures for patients with RA, with mean annual costs of $9,885 for medications alone [4].

Many correctional facilities maintain strict formularies that restrict access to expensive treatments, often requiring patients to fail multiple conventional therapies before qualifying for advanced agents [5]. While the federal 340B Drug Discount Program can help larger prison systems obtain reduced medication prices, many state and local jails lack the purchasing power to negotiate meaningful discounts [13]. This creates a scenario where clinically indicated therapy may be denied or discontinued based on cost rather than medical necessity.

The consequences of inadequate treatment

Patients with RA who do not achieve remission or low disease activity experience progressive joint destruction, functional decline, and increased cardiopulmonary risk [14]. Survey data from the National Rheumatoid Arthritis Society found that among patients not receiving advanced therapies, only 12.4% achieved a patient-acceptable symptom state, and 90% reported experiencing disease flares, with 23% experiencing more than six flares annually [15]. These patients also reported profound impacts on quality of life, with over 70% scoring in the high-severity range for sleep problems and fatigue [15].

RA-ILD represents a particularly devastating complication, affecting 10-30% of patients and carrying a median survival of 2 to 8 years [2]. The UIP pattern seen in our patient is associated with the worst prognosis among RA-ILD subtypes. While the relationship between disease activity control and ILD progression remains an area of active research, there is growing evidence that effective immunosuppression may stabilize pulmonary disease [8]. Our patient's clinical course, with respiratory deterioration occurring after discontinuation of effective therapy, underscores the importance of treatment continuity in patients with RA.

Additional barriers for incarcerated patients and potential solutions

Beyond medication access, incarcerated individuals with rheumatic diseases face numerous additional challenges. The prison environment itself can exacerbate disease through psychosocial stress, limited physical activity, poor sleep, and inadequate nutrition [5]. Depression, which negatively impacts rheumatic disease outcomes, affects up to 29% of prisoners [16]. Frequent transfers between facilities disrupt continuity of care and risk medication lapses [5]. Additionally, immunocompromised patients face heightened infection risks due to overcrowding, poor ventilation, and inadequate sanitation in many correctional facilities [5].

When catastrophic complications occur, incarcerated patients may face additional barriers to receiving advanced interventions. While our patient's ineligibility for ECMO and lung transplantation was primarily determined by his acute clinical status, his incarcerated status did factor into discussions regarding the feasibility of long-term post-transplant follow-up and compliance. These considerations, while clinically relevant, highlight how social determinants of health can compound medical challenges in ways that may disadvantage incarcerated patients even when overt discrimination is not present.

Addressing the healthcare needs of incarcerated individuals with rheumatic diseases requires systemic change. Terracina and Masurkar have proposed several solutions, including expanding telemedicine while ensuring in-person specialist access when needed, implementing standardized treatment protocols, providing specialized training for prison healthcare staff, expanding medication formularies, improving oversight of prison healthcare systems, and integrating electronic health records across facilities [5]. Additionally, policies enabling correctional facilities to access medications at reduced costs through programs similar to those available to other systems caring for low-income populations could improve access to essential therapies.

The rheumatology community has an important role to play in advocating for these changes and in developing guidelines specific to the unique challenges of correctional healthcare. Research is also needed to better understand the prevalence and outcomes of rheumatic diseases in incarcerated populations and to identify best practices for managing these conditions within the constraints of the correctional environment.

## Conclusions

This case highlights the profound challenges of managing rheumatoid arthritis in incarcerated populations and the importance of treatment continuity for patients with complex autoimmune diseases. While we cannot establish that continued tofacitinib therapy would have altered this patient's outcome, the temporal relationship between drug discontinuation and his fatal ILD exacerbation warrants reflection. As the incarcerated population ages and the prevalence of chronic rheumatic diseases increases, healthcare systems must grapple with how to provide adequate speciality care within the unique constraints of the correctional environment. This will require collaboration among policymakers, correctional healthcare administrators, and the rheumatology community to develop sustainable solutions that prioritize patient welfare while acknowledging resource limitations. Ultimately, the constitutional mandate to provide adequate medical care to incarcerated individuals must extend to complex chronic diseases like rheumatoid arthritis, including access to the advanced therapies that have transformed outcomes for patients fortunate enough to receive them.
